# P2X7 receptor antagonism by AZ10606120 significantly reduced in vitro tumour growth in human glioblastoma

**DOI:** 10.1038/s41598-023-35712-5

**Published:** 2023-05-24

**Authors:** Liyen K. Kan, Matthew Drill, Padmakrishnan C. Jayakrishnan, Richard P. Sequeira, Emily Galea, Marian Todaro, Paul G. Sanfilippo, Martin Hunn, David A. Williams, Terence J. O’Brien, Katharine J. Drummond, Mastura Monif

**Affiliations:** 1 Department of Neuroscience, Central Clinical School, Monash University, Melbourne, VIC Australia; 2 Department of Neurosurgery, The Alfred, Melbourne, VIC Australia; 3 Department of Physiology, The University of Melbourne, Melbourne, VIC Australia; 4 Department of Neurology, The Royal Melbourne Hospital, Melbourne, VIC Australia; 5 Department of Neurology, The Alfred, Melbourne, VIC Australia; 6 Department of Neurosurgery, The Royal Melbourne Hospital, Melbourne, VIC Australia

**Keywords:** Cancer, Immunology, Neuroscience

## Abstract

Glioblastomas are highly aggressive and deadly brain tumours, with a median survival time of 14–18 months post-diagnosis. Current treatment modalities are limited and only modestly increase survival time. Effective therapeutic alternatives are urgently needed. The purinergic P2X7 receptor (P2X7R) is activated within the glioblastoma microenvironment and evidence suggests it contributes to tumour growth. Studies have implicated P2X7R involvement in a range of neoplasms, including glioblastomas, although the roles of P2X7R in the tumour milieu remain unclear. Here, we report a trophic, tumour-promoting role of P2X7R activation in both patient-derived primary glioblastoma cultures and the U251 human glioblastoma cell line, and demonstrate its inhibition reduces tumour growth in vitro. Primary glioblastoma and U251 cell cultures were treated with the specific P2X7R antagonist, AZ10606120 (AZ), for 72 h. The effects of AZ treatment were also compared to cells treated with the current first-line chemotherapeutic drug, temozolomide (TMZ), and a combination of both AZ and TMZ. P2X7R antagonism by AZ significantly depleted glioblastoma cell numbers compared to untreated cells, in both primary glioblastoma and U251 cultures. Notably, AZ treatment was more effective at tumour cell killing than TMZ. No synergistic effect between AZ and TMZ was observed. AZ treatment also significantly increased lactate dehydrogenase release in primary glioblastoma cultures, suggesting AZ-induced cellular cytotoxicity. Our results reveal a trophic role of P2X7R in glioblastoma. Importantly, these data highlight the potential for P2X7R inhibition as a novel and effective alternative therapeutic approach for patients with lethal glioblastomas.

## Introduction

Brain cancer remains notoriously complex and difficult to treat. Gliomas account for approximately 80% of all brain malignancies^1^. Of these, a large proportion are glioblastomas, the most infiltrative and aggressive subtype. The median overall survival from glioblastoma diagnosis is estimated at 14–18 months^1^, with the 5-year survival rate of under 5%^2^. Disappointedly, survival rates for glioblastoma have not improved over the previous three decades^2^. This is largely due to ongoing limitations in the current treatment options. Despite progress in research on glioblastoma therapeutics, no new and more effective treatments have been translated into clinical care.

The current treatment regime for newly-diagnosed glioblastoma involves an initial maximal safe surgical resection, followed by concomitant radiotherapy and chemotherapy (temozolomide; TMZ) over 6 weeks, and subsequently six additional cycles of maintenance TMZ^3^. While treatment is multimodal, it serves only a modest prognostic benefit and is often associated with debilitating side-effects^3–13^. For example, TMZ chemotherapy has been shown to increase survival time by 2.5 months in some patients, with minimal therapeutic benefit in 60–75% of glioblastoma patients^4–6^. More effective and targeted therapies for gliomas are urgently needed.

A protein of interest in the tumour microenvironment is the P2X7 receptor (P2X7R)^14^. P2X7R is a trimeric transmembrane ion channel expressed on haematopoietic cells, including microglia, macrophages, and lymphocytes^15^. It binds to ATP and activates at ATP levels exceeding 200 micromolar. P2X7R can distinctly transition between two activation states: channel and pore. With transient ATP stimulation, the receptor is a highly selective ion channel that facilitates cationic movement across the plasma membrane. Prolonged exposure to ATP uniquely transforms P2X7R into a non-selective transmembrane pore that is also permeable to aqueous molecules of up to 900 Daltons^16–18^. The exact role of P2X7R in a physiological setting is unclear, but it is likely associated with cell trophism and cytokine release. Importantly, there are robust associations between P2X7R activation, inflammation, and immune modulation. P2X7R expression and activation has been further implicated in cellular energy metabolism and mitochondrial dysfunction^19,20^. The roles of P2X7R are likely diverse and its activation might induce either a growth-promoting or cytolytic effect in different pathological contexts.

P2X7R function has been implicated in various cancers, with studies demonstrating receptor involvement in melanoma^21^, uterine epithelial cancer^22^, leukaemia^23^ and lung cancer^24^. Importantly, in gliomas, P2X7R is expressed on both glioma cells and microglia, the main cellular infiltrates of the glioma microenvironment^14^. We have previously demonstrated P2X7R upregulation in gliomas^14^. In vitro studies have shown that P2X7R antagonism promoted the growth of glioma cells in the rat C6^25^ and mouse GL261^26^ glioma cell lines, suggesting a cytolytic role of P2X7R. In contrast, multiple studies have demonstrated reductions in glioma proliferation following P2X7R antagonism^27–30^, indicative of trophic P2X7R activity. A further study using rat C6 glioma cells demonstrated increased tumour cell motility following P2X7R stimulation^31^. Previous data from our laboratory on primary patient-derived glioblastoma cultures in vitro revealed a decrease in glioma cell count following P2X7R inhibition with Brilliant Blue G^14^. In a pilot study, we further demonstrated a significant reduction in glioblastoma cell numbers in both U251 human glioblastoma cells and primary patient-derived glioblastoma cultures following treatment with 5–25 μM of the specific P2X7R antagonist, AZ10606120 (AZ)^32^. Despite this, the precise functions of P2X7R in the glioma microenvironment remain unclear.

Here, we expand on our pilot data investigating the effect of P2X7R antagonism in both patient-derived glioblastoma samples and U251 human glioblastoma cells. Additionally, we compare the efficacy of P2X7R antagonism by AZ to that of the conventional chemotherapeutic drug, TMZ.

## Results

### P2X7R inhibition by AZ depletes tumour cells in a concentration-dependent manner in patient-derived primary glioblastoma samples

**Figure 1:**
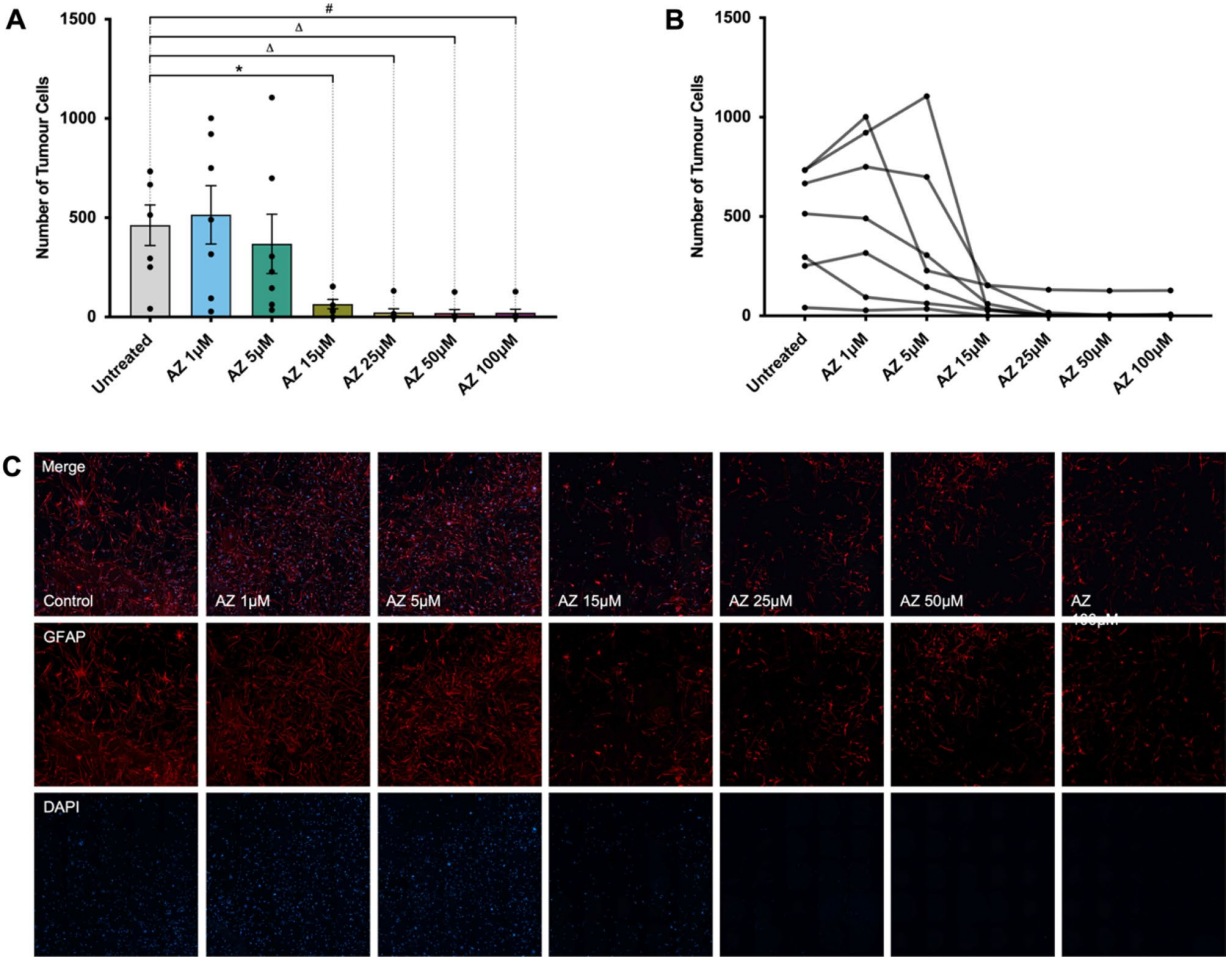
P2X7 receptor (P2X7R) antagonism by AZ10606120 (AZ) across AZ doses of 1–100 μM in human primary glioblastoma cultures. Glioblastoma specimens collected from patients undergoing routine tumour resection surgery were cultured and treated at 80% confluency with 1, 5, 15, 25, 50 or 100 μM of AZ for 72 h. The control group were untreated cells. Cells were fixed and stained with primary rabbit anti-glial fibrillary acidic protein (GFAP) antibody overnight at 4 °C, followed by secondary goat anti-rabbit antibody conjugated to Texas Red-X for 2 h at room temperature. All cell nuclei were counterstained with DAPI for 1 h at room temperature. Images were acquired with a Nikon Ti-E inverted fluorescence motorised microscope equipped with a sCMOS Andor Zyla camera at 20 × objective. The total number of GFAP + /DAPI + cells were quantified across 16 random fields for each sample and treatment group. (**A**) There was significant tumour cell depletion with AZ concentrations of 15 μM or greater. Results were yielded from a one-way repeated measures ANOVA with Tukey’s HSD post-hoc, F(1.61, 9.63) = 9.47, *p* = 0.007. N = 7 patients. Untreated versus AZ 15 μM: mean difference = 396.6 cells, 95% CI = 31.46 to 761.7; Untreated versus AZ 25 μM: mean difference = 439.0 cells, 95% CI = 45.49 to 832.5; Untreated versus AZ 50 μM: mean difference = 441.7 cells, 95% CI = 44.22 to 839.2; Untreated versus AZ 100 μM: mean difference = 441.0 cells, 95% CI = 42.21 to 839.8. *p = 0.04, Δ*p* = 0.031, #*p* = 0.032. Error bars represent SEM. (**B**) Individual patient responses to treatment with 1, 5, 15, 25, 50 and 100 μM AZ. (**C**) Image representation of primary glioblastoma cultures treated with various concentrations of AZ versus untreated control cells. GFAP and DAPI channels are red and blue, respectively.

In a previous pilot study, we demonstrated a significant decrease in tumour cell numbers in both U251 cells and primary patient-derived high-grade glioma cultures after treatment with varying doses of AZ for 72 h. We trialled concentrations of 5 and 25 μM for U251 cells and 15 μM for human gliomas, all three of which yielded a significantly lower glioma cell count compared to untreated cells^32^. Here, we further characterise the effects of AZ-mediated P2X7R antagonism in human glioblastomas. To establish an optimal AZ dosage regimen for treatment of primary patient-derived glioma cultures in vitro, cells at 80% confluency were treated with AZ concentrations of 1, 5, 15, 25, 50 and 100 μM for 72 h. Glial fibrillary acidic protein (GFAP) and DAPI double-positive cells were subsequently quantified for all treatment groups and untreated cells. In all patients, there was a significant reduction in tumour cell number following treatment with 15 μM AZ or greater, compared to untreated cells (mean GFAP + cells = 462.6 ± 102.4), with a mean total GFAP + cell count of 66 ± 23.51 for 15 μM AZ, 23.57 ± 18.19 for 25 μM AZ, 20.86 ± 17.72 for 50 μM AZ, and 21.57 ± 17.78 for 100 μM AZ (Fig. 1). There was no statistically significant difference in GFAP + cell count observed between AZ concentrations of 1 (515 ± 147.4 and 368.9 ± 148.9 cells) and 5 μM (368.9 ± 148.9 cells) versus untreated cells. We selected a dosage of 15 μM of AZ for use in subsequent experiments, given that it was the lowest concentration demonstrating effective tumour cell depletion.

### P2X7R inhibition by AZ and its comparison to TMZ chemotherapy

**Figure 2:**
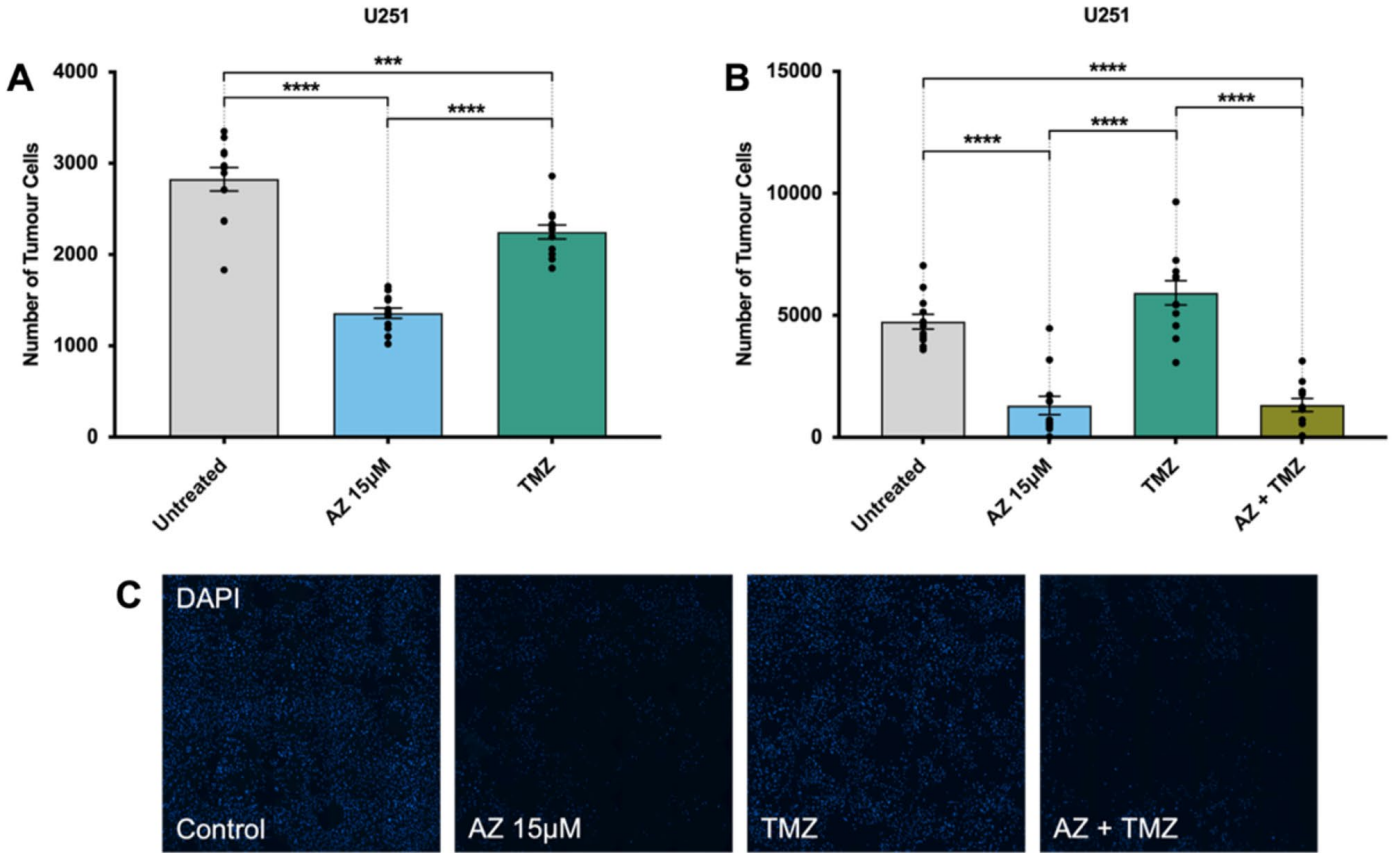
The effects of P2X7 receptor (P2X7R) antagonism by AZ10606120 (AZ) compared to conventional temozolomide (TMZ) chemotherapy on the U251 human glioblastoma cell line. U251 human glioblastoma cells were cultured and treated at 80% confluency with 15 μM AZ, 50 μM TMZ and a combination of AZ + TMZ for 72 h. The control group were untreated cells. U251 cultures were counterstained with DAPI for 1 h at room temperature. Images were acquired with a Nikon Ti-E inverted fluorescence motorised microscope equipped with a sCMOS Andor Zyla camera at 20 × objective. The total number of DAPI + cells were quantified across 16 random fields for each sample and treatment group. (**A**) Comparison of AZ 15 μM with TMZ in U251 cells. Results were yielded from a one-way ANOVA with Tukey’s HSD post-hoc, F(2, 33) = 64.87, *p* < 0.0001. N = 12 replicates. Untreated versus TMZ: mean difference = 578.3 cells, 95% CI = 259.3 to 897.4; Untreated versus AZ 15 μM: mean difference = 1470 cells, 95% CI = 1151 to 1789; AZ 15 μM versus TMZ: mean difference = − 891.5, 95% CI = − 1211 to − 572.5. ***p = 0.0003, ****p < 0.0001. Error bars represent SEM. (**B**) Comparison of 15 μM AZ and AZ-TMZ co-therapy with TMZ in U251 cells. Results were yielded from a one-way ANOVA with Tukey’s HSD post-hoc. N = 12 replicates, F(3, 44) = 40.77, *p* < 0.0001. Untreated versus AZ 15 μM: mean difference = 3435 cells, 95% CI = 2035 to 4835; Untreated versus TMZ: mean difference = − 1177 cells, 95% CI = − 2577 to 223.2, *p* = 0.13; Untreated versus AZ + TMZ: mean difference = 3419 cells, 95% CI = 2019 to 4819; AZ 15 μM versus TMZ: mean difference = − 4612 cells, 95% CI = − 6012 to − 3212. AZ 15 μM versus AZ + TMZ: mean difference = − 16.33 cells, 95% CI = − 1416 to 1384, *p* > 0.99; TMZ versus AZ + TMZ: mean difference = 4596 cells, 95% CI = 3196 to 5996. *p < 0.0001. Error bars represent SEM. (**C**) Image representation of U251 cells treated with 15 μM AZ, TMZ, AZ + TMZ and control.

We have previously demonstrated significant reductions in tumour cell number after treatment with AZ on both U251 human glioblastoma cells and surgically resected human glioma samples^32^. Extending from these results, our next aim was to elucidate the anti-tumour activity of 15 μM of AZ, in comparison to the most common conventional chemotherapy agent, TMZ. Cells were treated for 72 h with either AZ (15 μM) or TMZ (50 μM). Following treatment, cells were fixed, stained and the number of DAPI + cells for U251 cells, and GFAP + /DAPI + cells for primary glioblastoma cultures were quantified via fluorescence microscopy. We conducted an initial experiment on U251 cells comparing AZ 15 μM to TMZ alone. Both TMZ and AZ depleted U251 cell numbers (2249 ± 77.75 and 1357 ± 56.42 cells, respectively) when compared to untreated cells (2827 ± 127 cells) (Fig. 2A). Interestingly, cells treated with AZ had a significantly lower U251 cell count than TMZ-treated cells (Fig. 2A). Similarly, in a subsequent experiment, treatment with AZ significantly reduced U251 cell count (1309 ± 378.3 cells) compared to both untreated cells (4744 ± 302.4 cells) and TMZ-treated cells (5921 ± 495.1 cells) (Fig. 2B). However, there was no significant tumour inhibition observed with TMZ treatment (Fig. 2B). The effects of AZ and TMZ treatment were also tested at 24 and 48 h post-treatment, but showed no statistically significant difference in tumour cell number compared to untreated cells (Supplementary Fig. 1).

**Figure 3:**
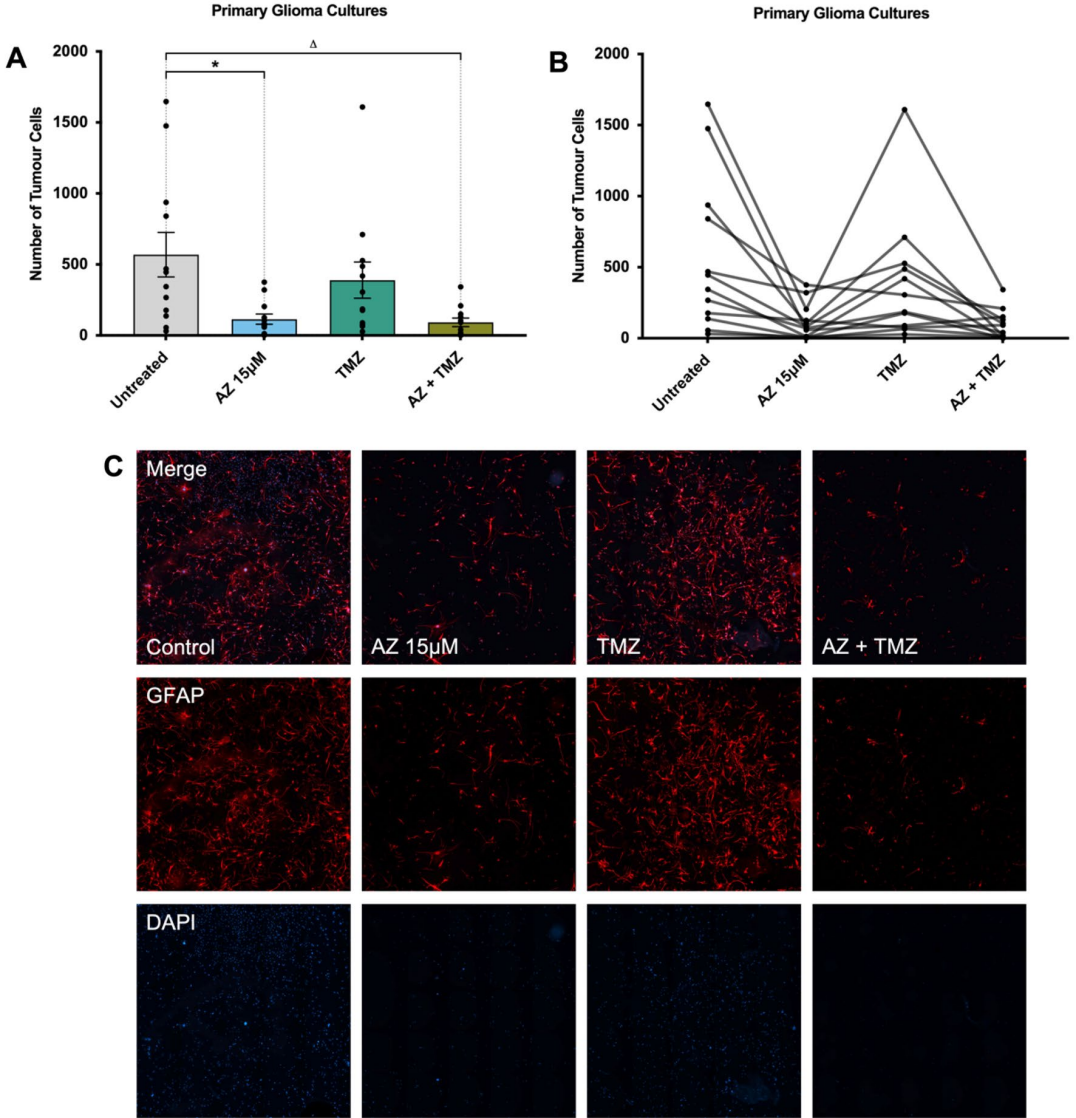
The effects of P2X7 receptor (P2X7R) antagonism by AZ10606120 (AZ) compared to conventional temozolomide (TMZ) chemotherapy on patient-derived primary glioblastoma cultures. Glioblastoma specimens collected from patients undergoing routine tumour resection surgery were cultured and treated at 80% confluency with 15 μM AZ, 50 μM TMZ and a combination of AZ + TMZ for 72 h. The control group were untreated cells. Primary glioblastoma cultures were fixed and stained with primary rabbit anti-glial fibrillary acidic protein (GFAP) antibody overnight at 4 °C, followed by secondary goat anti-rabbit antibody conjugated to Texas Red-X for 2 h at room temperature. Cells were counterstained with DAPI for 1 h at room temperature. Images were acquired with a Nikon Ti-E inverted fluorescence motorised microscope equipped with a sCMOS Andor Zyla camera at 20 × objective. The total number of GFAP + /DAPI + cells were quantified across 16 random fields for each sample and treatment group. (**A**) Comparison of AZ 15 μM and AZ-TMZ co-therapy with TMZ in primary glioblastoma cultures. Results were yielded from a one-way repeated measures ANOVA with Tukey’s HSD post-hoc, F(1.81, 19.9) = 7.79, *p* = 0.004. N = 12 patients. Untreated versus AZ 15 μM: mean difference = 454.8 cells, 95% CI = 13.05 to 896.4, *p = 0.04; Untreated versus TMZ: mean difference = 179.3 cells, 95% CI = − 182.2 to 540.7, *p* = 0.47; Untreated versus AZ + TMZ: mean difference = 476.7 cells, 95% CI = 69.78 to 883.6, Δ*p* = 0.02; AZ 15 μM versus TMZ: mean difference = − 275.5 cells, 95% CI = − 632.5 to 81.46, *p* = 0.15; AZ 15 μM versus AZ + TMZ: mean difference = 21.92 cells, 95% CI = − 59.3 to 103.1, *p* = 0.85; TMZ versus AZ + TMZ: mean difference = 297.4 cells, 95% CI = − 31.45 to 626.3, *p* = 0.08. Error bars represent SEM. (**B**) Individual patient responses to treatment with AZ 15 μM, TMZ and AZ + TMZ. (**C**) Image representation of primary glioblastoma cultures treated with AZ 15 μM, TMZ, AZ + TMZ and control. GFAP and DAPI channels are red and blue, respectively.

In primary glioblastoma cultures, treatment with 15 μM of AZ also significantly reduced GFAP + cell numbers (114.3 ± 35.9 vs. 569.1 ± 157 cells in AZ and untreated groups, respectively), although there were no significant differences between both the untreated versus TMZ group and the AZ versus TMZ group (Fig. 3A). Of note, there was variability in patient response to TMZ (Fig. 3B), which likely affected the differences in cell count observed between TMZ and treatment groups. Nonetheless, the above data collectively demonstrated a significant reduction in tumour cell count in both U251 cells and human glioblastoma samples following P2X7R antagonism with 15 μM of AZ, supportive of a P2X7R-mediated anti-tumour effect. Additionally, AZ treatment shows potential as either an adjunctive therapy or a more effective treatment alternative to TMZ in U251 cells, although this should be explored further with human glioblastoma samples.

### Co-administration of AZ with TMZ does not synergistically deplete tumour cell number in U251 cells and primary patient-derived glioblastoma cultures

We sought to additionally investigate the effect of AZ and TMZ co-therapy and whether these reagents would synergistically inhibit tumour proliferation. U251 cells were treated with a combination of 15 μM of AZ and 50 μM of TMZ for 72 h. The effect of AZ and TMZ co-therapy was also compared to that of untreated cells, and cells treated with either AZ or TMZ alone. Cells were subsequently fixed, stained and imaged via fluorescence microscopy. DAPI + and GFAP + /DAPI + cells were quantified for U251 cells and primary human glioblastoma cultures, respectively. For U251 cells, a one-way ANOVA yielded a statistically significant difference between groups. Notably, there was no significant difference in U251 cell count between cells treated with AZ and TMZ in combination (1326 ± 265 cells) and cells treated with AZ alone (1309 ± 378.3 cells) (Fig. 2B). Both AZ-TMZ co-treated cells and AZ treated cells had significantly lower tumour cell numbers versus untreated cells (4744 ± 302.4 cells) and TMZ-treated cells (5921 ± 495.1 cells) (Fig. 2B).

Similarly, in primary human glioblastoma cultures, there was no significant difference in tumour cell count between AZ-treated (114.3 ± 35.9 cells) and AZ-TMZ co-treated cells (92.42 ± 30.06 cells) (Fig. 3A). Lower tumour cell numbers were observed between both cells treated with AZ and AZ-TMZ, and untreated cells (569.1 ± 157 cells) (Fig. 3A). No statistically significant differences were observed between the AZ and AZ-TMZ groups and TMZ-treated cells, despite the presence of a trend demonstrating increased tumour cell depletion in AZ and AZ-TMZ treated groups.

**Figure 4:**
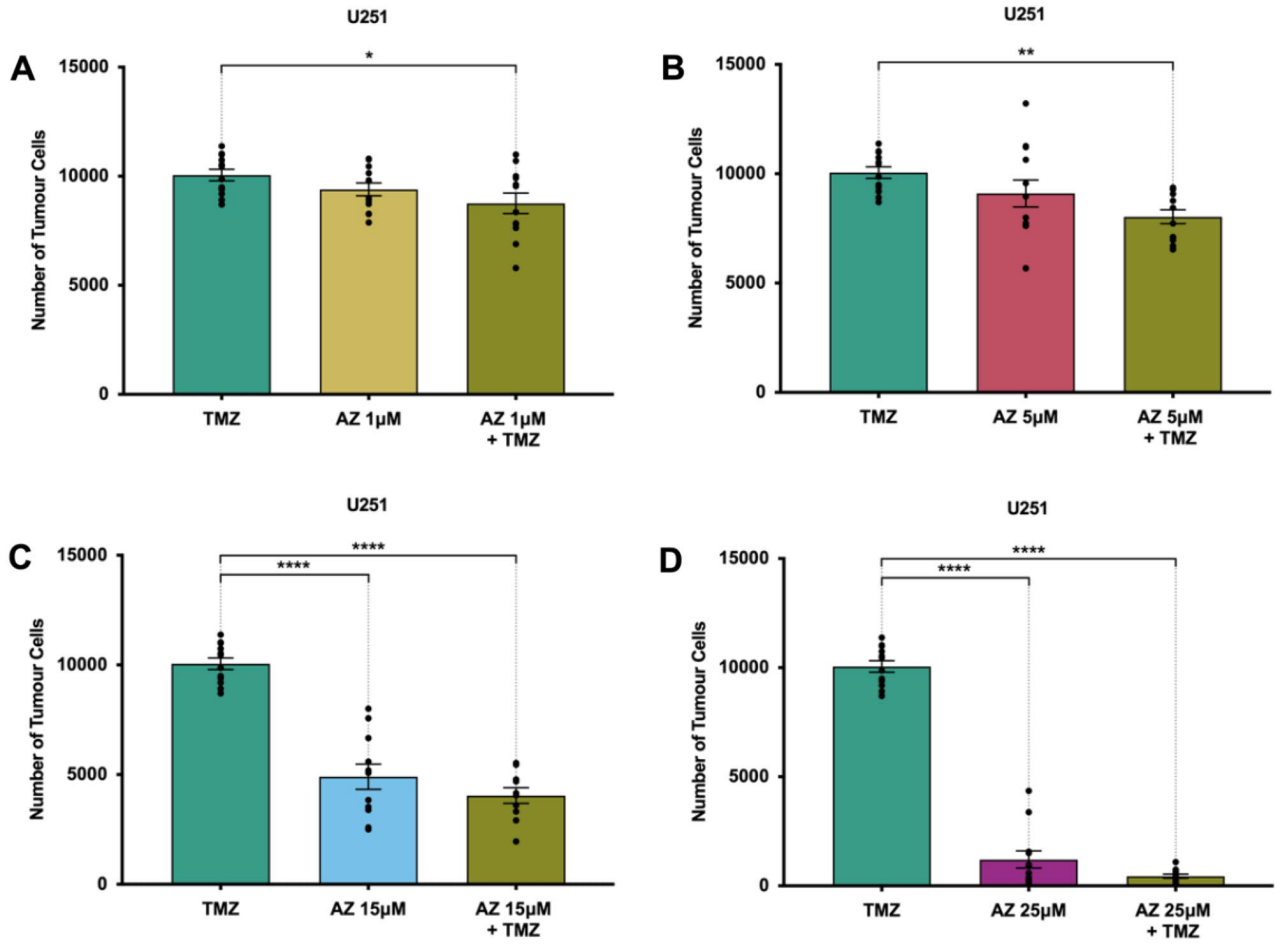
Co-treatment of AZ10606120 (AZ) and temozolomide (TMZ) in U251 cells. U251 human glioblastoma cells were cultured and treated at 80% confluency with various concentrations of AZ, 50 μM TMZ and a combination of AZ + TMZ for 72 h. Cells were fixed and stained with DAPI for 1 h at room temperature. The total number of DAPI + cells were quantified across 16 random fields for each sample and treatment group. (**A**) Comparison of AZ, TMZ and AZ + TMZ with AZ 1 μM in U251 cells. Results were yielded from a one-way ANOVA with Tukey’s HSD post-hoc, F(2, 33) = 3.77, *p* = 0.03. N = 12 replicates. TMZ versus AZ 1 μM: mean difference = 659.8 cells, 95% CI = − 562.5 to 1882, *p* = 0.39; TMZ versus AZ 1 μM + TMZ: mean difference = 1299 cells, 95% CI = 76.46 to 2521, *p = 0.04; AZ 1 μM versus AZ 1 μM + TMZ: mean difference = 639 cells, 95% CI = − 583.4 to 1861, *p* = 0.41. (**B**) Comparison of AZ, TMZ and AZ + TMZ with AZ 5 μM in U251 cells. Results were yielded from a one-way ANOVA with Tukey’s HSD post-hoc, F(2, 33) = 4.05, *p* = 0.03. N = 12 replicates. TMZ versus AZ 5 μM: mean difference = 958.9 cells, 95% CI = − 528.6 to 2446, *p* = 0.27; TMZ versus AZ 5 μM + TMZ: mean difference = 2024 cells, 95% CI = 536 to 3511, **p = 0.006; AZ 5 μM versus AZ 5 μM + TMZ: mean difference = 1065 cells, 95% CI = − 422.9 to 2552, *p* = 0.20. (**C**) Comparison of AZ, TMZ and AZ + TMZ with AZ 15 μM in U251 cells. Results were yielded from a one-way repeated measures ANOVA with Tukey’s HSD post-hoc, F(2, 30) = 3.72. N = 12 patients. TMZ versus AZ 15 μM: mean difference = 5152 cells, 95% CI = 3728 to 6576; TMZ versus AZ 15 μM + TMZ: mean difference = 6013 cells, 95% CI = 4552 to 7474; AZ 15 μM versus AZ 15 μM + TMZ: mean difference = 860.7 cells, 95% CI = − 629.9 to 2351, *p* = 0.34. ****p < 0.0001. (**D**) Comparison of AZ, TMZ and AZ + TMZ with AZ 25 μM in U251 cells. Results were yielded from a one-way repeated measures ANOVA with Tukey’s HSD post-hoc, F(2, 33) = 3.31, *p* = 0.049. N = 12 patients. TMZ versus AZ 25 μM: mean difference = 8848 cells, 95% CI = 7891 to 9805; TMZ versus AZ 25 μM + TMZ: mean difference = 9609 cells, 95% CI = 8652 to 10,567; AZ 25 μM versus AZ 25 μM + TMZ: mean difference = 761.4 cells, 95% CI = − 196.1 to 1719, *p* = 0.14; ****p < 0.0001. Error bars represent SEM.

There was no synergistic anti-tumour effect observed with AZ-TMZ co-administration. We postulated that this result could have been due to the limited bandwidth available for synergistic activity to occur, given that 15 μM of AZ was shown to reduce glioblastoma cell numbers with high efficacy. With low cell numbers in cultures treated with AZ 15 μM, AZ-TMZ combination therapy may not exert a discernible effect; a synergistic effect might hence be observable with lower AZ concentrations. To explore this, we co-treated U251 cells with varying AZ concentrations (1 μM, 5 μM, 15 μM and 25 μM) and TMZ, and compared the effects to both TMZ alone, AZ alone and untreated cells. No statistically significant difference in tumour cell count was detected between U251 cells treated with AZ-TMZ co-therapy and AZ alone, for all AZ concentrations (Fig. 4). However, there was a statistically significant decrease in tumour cell number in AZ-TMZ co-treated cells for both AZ 1 μM (9399 ± 295.8 cells) and AZ 5 μM (9100 ± 615 cells) compared to TMZ (10,059 ± 262.5 cells), despite no significant changes in cell count between AZ alone and TMZ (Fig. 4A, B). Given that there was no observed difference in U251 cell count between AZ and AZ-TMZ treated groups, this data is not supportive of AZ-TMZ mediated synergy. However, this requires further clarification. Nonetheless, our results demonstrate improved tumour-depleting activity by AZ over TMZ, with no synergistic anti-tumour effect observed between AZ and TMZ.

### AZ increases lactate dehydrogenase (LDH) release in human tumour samples

**Figure 5:**
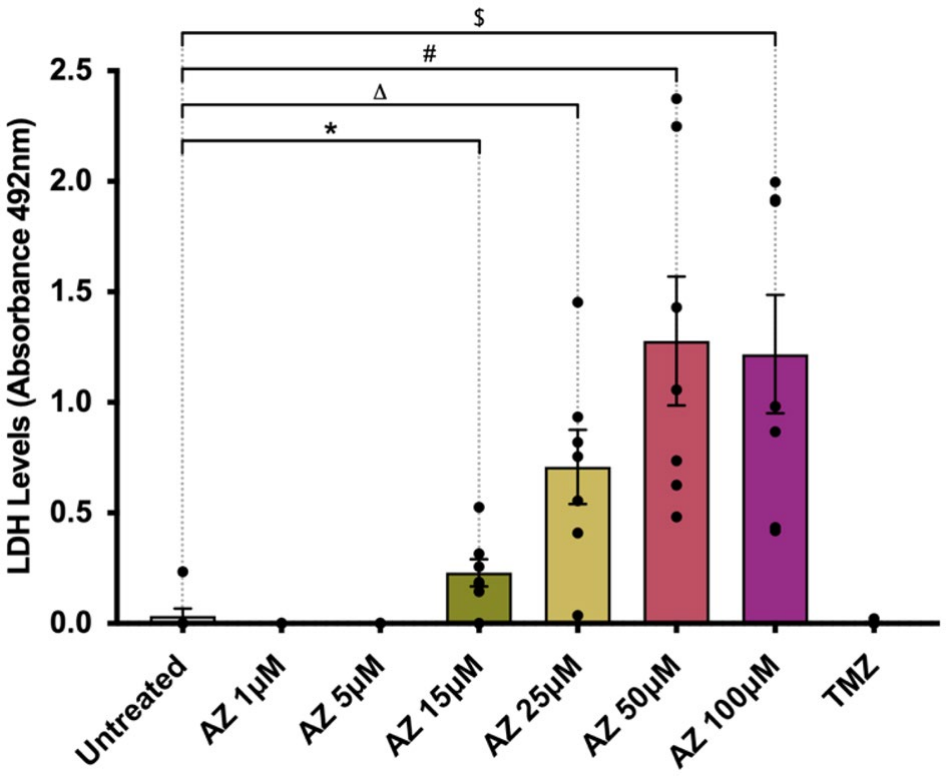
Lactate dehydrogenase (LDH) levels in human primary glioblastoma cultures following P2X7 receptor (P2X7R) antagonism with AZ10606120 (AZ). Glioblastoma specimens collected from patients undergoing routine tumour resection surgery were cultured and treated at 80% confluency with 1, 5, 15, 25, 50 or 100 μM of AZ for 72 h. As a comparison, cells were also treated with 50 μM of temozolomide (TMZ), the conventional chemotherapeutic drug. The control group were untreated cells. Culture supernatants were processed and utilised in an LDH cytotoxicity assay. Supernatant LDH levels are proportional to the absorbance at 492 nm. Treatment with AZ concentrations of 15 μM or greater significantly increased LDH release by primary human glioblastoma cells. Results were yielded from a one-way repeated measures ANOVA with Dunnett’s post-hoc, F(1.48, 8.87) = 16.93, *p* = 0.002. N = 7 patients. Untreated versus AZ 1 μM: mean difference = 0.03, 95% CI = − 0.09 to 0.15, *p* = 0.84; Untreated versus AZ 5 μM: mean difference = 0.03, 95% CI = − 0.09 to 0.15, *p* = 0.84; Untreated versus AZ 15 μM: mean difference = − 0.20, 95% CI = − 0.34 to − 0.05, *p = 0.01; Untreated versus AZ 25 μM: mean difference = − 0.68, 95% CI = − 1.26 to − 0.09, Δ*p* = 0.03; Untreated versus AZ 50 μM: mean difference = − 1.25, 95% CI = − 2.28 to − 0.21, #*p* = 0.02; Untreated versus AZ 100 μM: mean difference = − 1.18, 95% CI = − 2.09 to − 0.28, $*p* = 0.015; Untreated versus TMZ: mean difference = 0.03, 95% CI = − 0.09 to 0.15, *p* = 0.90. Error bars represent SEM.

Treatment with AZ resulted in significantly lower glioblastoma cell counts. To determine whether this anti-tumour effect was due to plasma membrane damage, we conducted a lactate-dehydrogenase (LDH) cytotoxicity assay to assess the LDH levels present in primary glioblastoma culture supernatants. Glioblastoma cultures were treated at 80% confluency with 1 μM, 5 μM, 15 μM, 25 μM, 50 μM or 100 μM of AZ for 72 h. LDH levels in culture supernatants were subsequently quantified and are directly proportional to absorbance levels at 492 nm. Notably, there was a significantly higher level of LDH in the culture supernatants of cells treated with 15 μM of AZ onwards, compared to the untreated control (Fig. 5). There was also a trend for increasing supernatant LDH levels with increasing AZ concentration. Treatment with TMZ did not induce LDH release in patient-derived glioblastoma cultures (Fig. 5). From this data, AZ treatment likely induces a cytotoxic membrane-damaging effect variable to TMZ, although the exact mode of cell death requires further investigation.

## Discussion

Glioblastomas are highly invasive and serve as the third leading cause of cancer-related death in young people aged 15 to 24. Importantly, despite advances in treatment, no improvement in glioma survival rates have been observed across the past three decades^2^. Here, we investigated the effect of P2X7R antagonism by AZ in both human U251 glioblastoma cells—which are devoid of immune cells—and primary patient-derived glioblastoma cultures. Importantly, treatment with AZ for 72 h significantly depleted tumour cells in vitro, in both primary patient-derived glioblastoma cultures and U251 glioblastoma cells. AZ treatment was also more effective than conventional TMZ chemotherapy, although no synergistic anti-tumour effect was observed between AZ and TMZ. LDH levels were significantly increased following AZ treatment, indicative of cellular cytotoxicity, but more clarification of the mode of tumour cell death induced by AZ treatment is required. Nonetheless, this study highlights the potential for P2X7R inhibition to serve as a novel therapeutic for glioblastoma, superior to conventional TMZ.

An earlier study conducted by our laboratory was one of the first to trial the effects of AZ treatment in U251 cells and human glioma samples^32^. This dataset only included a dosage of 5 and 25 μM of AZ for U251 cells and 15 μM for human glioma samples. A dose–response experiment of AZ in U251 cells was subsequently conducted by our laboratory and established a half maximal inhibitory (IC50) concentration of 17 μM (Drill et al. Unpublished data). In this study, we sought to establish the lowest effective treatment concentration of AZ in primary human glioblastoma cultures for use in subsequent cell quantification and cytotoxicity experiments. Across a 72-h treatment period, we observed a significant reduction in tumour cell numbers in tumours treated with 15 μM of AZ and above (Fig. 1). There was also a trend for increasing tumour inhibition with increasing doses of AZ (Fig. 1). A concentration of 15 μM was the lowest effective AZ dosage for human glioblastoma samples. AZ dosage in the current literature is variable, with previous studies using concentrations equivalent to 0.3 μM^33^, 10 μM^34^, 100 μM or 200 μM^35^. However, these studies were conducted on models of mammary and pancreatic cancer^33–35^, and not glioma.

Importantly, treatment with 15 μM of AZ at 72 h significantly depleted tumour cell numbers in both U251 cells and human glioblastoma samples (Fig. 2, Supplementary Fig. 1). In U251 cells, there were significantly lower numbers of tumour cells in cultures treated with AZ, compared to those treated with TMZ, across two experiments (Fig. 2). However, in patient glioblastoma cultures, no significant changes in tumour cell numbers were observed between the same groups, although an overall trend for higher efficacy of AZ over TMZ was speculated (Fig. 3A). There was notable variability in patient response to TMZ, which might have affected the overall statistical differences observed between the two groups (Fig. 3B). Variability in patient response to TMZ therapy is well-documented. While the drug generally increases overall survival by 2.5 months^4^, around 60–75% of glioblastoma patients receive no therapeutic benefit due to genetic or acquired TMZ resistance^5,6,36^. In this study, specimens were collected from both newly diagnosed and recurrent glioblastoma patients, so it is not surprising that TMZ resistance was observed in some samples. Of note, O6-methylguanine methyltransferase (MGMT) promoter methylation status, commonly linked to differential responses to TMZ^37^, was not established in this study. There was also evidence of acquired TMZ resistance in U251 cells. Initially, there was a minor anti-tumour effect with TMZ administration (Fig. 2A), which diminished in a subsequent experiment with later U251 passages (Fig. 2B). While the U251 cell line is known to be sensitive to TMZ^37^, there is documentation of acquired TMZ resistance in U251 cells likely due to mismatch repair deficiency^38^. We observed no statistically significant tumour-killing activity with AZ treatment after 24 or 48 h (Supplementary Fig. 1). There is likely a time-dependent effect of AZ, influenced by factors including threshold cellular AZ concentration and differential sensitivity to AZ at various cell-cycle stages. Nonetheless, in this study all U251 cells and patients appeared to have responded well to AZ treatment after 72 h (Figs. 2, 3). Indeed, the anti-tumour effect of AZ has been implicated in other neoplastic diseases, but limited studies have elucidated its role in glioblastomas. In a neuroblastoma mouse model, P2X7R inhibition with AZ resulted in a significant reduction in VEGF secretion, subsequently hindering blood vessel formation required for angiogenesis^39^. AZ treatment also inhibited tumour cell migration and invasion and reduced the fibrous stroma in pancreatic cancer^40^. AZ administration further inhibited mesothelioma growth in both in vitro and in vivo experiments^41^. Previous work from our laboratory revealed increased expression of granulocyte–macrophage colony-stimulating factor (GM-CSF) in U251 cells following treatment with AZ^42^. In glioblastomas, GM-CSF promotes immunosuppression by favouring a shift toward granulocytic cells, rather than lymphocytic lineages, during haematopoiesis^43^. GM-CSF upregulation also induces overexpression of IL-4 receptor-α on myeloid-derived suppressor cells, which facilitates immunosuppression^44^. Few other studies have investigated the effects of P2X7R inhibition by AZ in human gliomas. The results of this current study support a trophic role of P2X7R in glioblastoma, where its inhibition by AZ exerts a largely anti-tumour effect.

We also wanted to determine whether co-treatment with AZ and TMZ fosters a synergistic anti-tumour response. No synergistic effect was observed with AZ-TMZ co-administration at AZ concentrations of 1 μM, 5 μM, 15 μM and 25 μM in U251 cells (Fig. 2B, 4). We initially assessed for AZ-TMZ synergy with 15 μM of AZ but postulated that the lack of effect could have been due to the massive tumour-depleting activity of AZ 15 μM, limiting the bandwidth for an observable synergistic effect of both drugs to occur. However, given that no significantly enhanced reductions in tumour cell numbers were observed between U251 cultures treated with AZ (1 and 5 μM) and AZ-TMZ co-therapy, it is likely that both drugs do not act in synergy. It is possible that the TMZ concentration of 50 μM was not sufficient to induce an ant-tumour effect, and thus the reductions in cell count in groups treated with AZ and TMZ were solely due to AZ activity. TMZ dosages for in vitro studies have been controversial. The current standard of care incorporates maintenance TMZ at a dose of 150–200 mg/m^2^ on days 1 to 5 of a 28-day cycle^6^, with peak concentrations of the drug reaching 30–50 μM in tumour and plasma^45^. We selected the TMZ dosage based on this data. However, within the literature, there are discrepancies with determining optimal clinically relevant dosages. For example, other studies have suggested optimal TMZ doses of 0.82–9.94 μM^46^, 1.55–4.64 μM^47^ and 14.94–34.51 μM^48^, based on peak drug concentrations in either tumour, peri-tumour or cerebrospinal fluid. Treatment length might have further affected TMZ efficacy, although multiple studies have utilised the 72-h timeframe with observed efficacy^49–52^. It is important to note that this is not representative of the clinical TMZ treatment regimen, which involves cyclic, long-term TMZ use; a single high dose of TMZ is likely to have diminished efficacy over continued usage^36^. Clinically relevant drug doses are difficult to replicate in in vitro experiments, which serves as a major limitation of this study. Nonetheless, here, TMZ was ineffective in reducing tumour growth in primary glioblastoma cultures and did not act in synergy with various concentrations of AZ.

Tumour inhibition by P2X7R antagonism is likely governed by a multitude of downstream mediators. Our data support a tumour depleting role of AZ treatment, but it is currently unclear how the compound exerts its anti-tumour activity. As a first step, we explored whether AZ was causing cytotoxicity via plasma membrane damage, through the release of LDH in patient samples. LDH is a ubiquitous cytoplasmic enzyme secreted into the extracellular space upon plasma membrane damage. Its release into cell culture supernatants has been widely utilised as a measure of cellular damage, particularly of cells undergoing apoptosis and necrosis^53^. We observed a significant increase in supernatant LDH levels following treatment with AZ at 15 μM onwards, with a trend showing increasing LDH release with increasing AZ dosage (Fig. 5). In contrast, TMZ did not promote LDH release in patient glioblastoma samples. P2X7R blockade by AZ has been weakly linked to LDH release, although studies are limited and present conflicting results. For example, in immortalised malignant pleural mesothelioma cell lines, receptor antagonism by AZ increased LDH release^40^, whereas pre-treatment with AZ prevented the ATP-mediated release of LDH in the J774 macrophage cell line^54^. The latter study also observed P2X7-mediated IL-1β release in LPS-stimulated macrophages, which was associated with a small amount of LDH release^54^. Nonetheless, in the present study, the trend displaying increasing LDH release coupled with decreasing tumour cell numbers with increasing AZ dosage is suggestive of AZ-mediated cytotoxicity.

The mechanisms of tumour inhibition by P2X7R are likely multifaceted, involving crosstalk between glioblastoma cells, immune mediators and other components of the tumour microenvironment. P2X7R blockade likely hinders NLRP3 inflammasome-controlled release of tumour-promoting mediators such as IL-1β. For example, since the P2X7R pore mediates IL-1β release^55^, inhibiting receptor activity is likely to greatly diminish IL-1β levels within the tumour microenvironment leading to reduced overall immunosuppression. IL-1β is known to fuel microglial polarisation into a trophic, immunosuppressive phenotype ideal for glioma growth^55,56^. The cytokine also directly acts on glioma cells by increasing tumour cell migration and invasion^57^. Moreover, halting P2X7R activity might induce metabolic dysfunction of tumour cells directly, leading to tumour cell death and hence glioma inhibition. P2X7R has been shown to increase cellular lactate output and glycogen storage, as well as upregulate glycolytic enzymes, enhancing the ability of tumour cells to adapt to unfavourable ambient conditions^58^. Enhanced mitochondrial depolarisation has also been observed in C6 glioma cells following P2X7R activation^59^. There seems to be a clear metabolic association with P2X7R stimulation. Interestingly, receptor stimulation has been shown to assist in the maintenance of cancer stem cells^60,61^, which are key contributors to treatment resistance and tumour recurrence^62^. Hence, P2X7R antagonism might also enhance tumour killing by preventing cancer stem cell development. Investigating the efficacy of AZ treatment in glioblastoma stem cells—particularly three-dimensional glioblastoma cell culture systems^63^—would be pivotal, given that an ideal therapeutic would also be effective against cells that are resistant to conventional treatment.

Collectively, these experiments suggest a basal cancer-promoting role of P2X7R activation in the glioblastoma setting. P2X7R antagonism by AZ inhibits tumourigenesis with high efficacy. The downstream tumour-inhibiting mechanisms of P2X7R blockade by AZ are still unclear, but results from this study support cytotoxic P2X7R-mediated anti-tumour activity facilitated by glioblastoma cells alone (evidenced by U251 cells), in addition to potential downstream immunological responses within the wider tumour microenvironment (evidenced by primary human glioblastoma cultures). There was no synergistic anti-tumour effect with AZ-TMZ combination therapy. However, AZ treatment appeared to be more effective than conventional TMZ chemotherapy. The next stage is to illuminate potential downstream responses of P2X7R inhibition, particularly in terms of immunological and metabolic mediators. Delineating associations between P2X7R expression and various pro-tumour, anti-tumour, and microenvironmental components would be a valuable first step. Furthermore, repeating these experiments on glioblastoma cancer stem cells is necessary to test the efficacy of AZ in the context of treatment resistance and tumour recurrence. Nonetheless, P2X7R inhibition significantly depleted glioblastoma cells in U251 cells and patient glioblastoma samples. This potentiates P2X7R as an ideal new therapeutic target for aggressive brain cancer. Importantly, AZ shows promise as an alternative or adjunctive therapy for glioblastoma.

## Materials and methods

### Patient-derived primary glioblastoma culture

Human glioblastoma samples were collected from patients undergoing routine tumour resection surgery by a qualified neurosurgeon at The Royal Melbourne Hospital and The Alfred, as part of the Alfred Brain Tumour Biobank (Melbourne, Australia). Protocols for obtaining and handling human brain tissue were reviewed and approved by the Human Research Ethics Committees (HREC) of the RMH (HREC 2006.119) and The Alfred (HREC 2017.450). All experiments were performed in accordance with relevant guidelines and regulations. Written informed consent was obtained from all patients or respective legally authorised representatives. Upon resection, tumour samples were fragmented and immersed in Earle’s Balanced Salt Solution (EBSS; Thermo Fisher Scientific Australia, cat no. 14155063) containing papain (200 units; Merck Australia, cat no. P3125) at 37 °C for 35 min. Digested tissue samples were then washed in triplicate with culture media—Minimum Essential Medium (MEM; Thermo Fisher Scientific Australia, cat no. 10370021) supplemented with 1 mM D-glucose (Merck Australia, cat no. G7021), 2 mM L-glutamine (Merck Australia, cat no. G7513), 50 units/mL penicillin–streptomycin (Thermo Fisher Scientific Australia, cat no. 15070063), 10% heat inactivated foetal bovine serum (FBS; Thermo Fisher Scientific Australia, cat no. 10100147) and Corning® MITO + Serum Extender (Merck Australia, cat no. DLW355006). Cells were homogenised and seeded at approximately 1 × 10^5^ cells per well into 12-well cell culture plates (Merck Australia, cat no. SIAL0512) containing 18 mm round glass coverslips (Thermo Fisher Scientific Australia). Coverslips were pre-coated with poly-D-lysine hydrobromide (PDL; Merck Australia, cat no. P6407). Primary glioblastoma cultures were maintained in a humidified incubator at 37 °C with 5% CO_2_.

### U251 glioblastoma cell culture

The U-251 MG (U251) human glioblastoma cell line was obtained from Merck Australia (cat no. 09063001, RRID: CVCL_0021). U251 cells were cultured in Dulbecco’s Modified Eagle’s Medium (DMEM; Lonza, cat no. 12-604F) supplemented with 1 mM sodium pyruvate (Thermo Fisher Scientific Australia, cat no. 11360070), 50 units/mL penicillin–streptomycin, 10% FBS and 1% non-essential amino acids (NEAA; Thermo Fisher Scientific Australia, cat no. 11140050). Cells were maintained in a humidified incubator at 37 °C with 5% CO_2_ and passaged routinely at 80% confluency.

### Treatment reagents

Primary human glioblastoma cultures and U251 cultures were treated at 80% confluency with the P2X7R antagonist, AZ106061206 (AZ; Tocris Biosciences, cat no. 3323), and/or the conventional chemotherapy drug, temozolomide (TMZ; Merck Australia, cat no. T2577), for 72 h. To compare the effects of various treatment lengths, U251 cells were also treated with AZ and/or TMZ for 24 h and 48 h. Both AZ and TMZ were dissolved in dimethyl sulfoxide (DMSO; Merck Australia, cat no. W387509) prior to use according to manufacturers’ instructions. A TMZ concentration of 50 μM was utilised for all experiments involving TMZ treatment, based on previous studies demonstrating peak physiological TMZ concentrations of 30–50 μM in both tumour and plasma of patients undergoing TMZ maintenance therapy for glioblastoma^45^. The following concentrations of AZ were used: 1 μM, 5 μM, 15 μM, 25 μM, 50 μM and 100 μM.

### Cell staining

Following treatment, primary human glioblastoma cultures and U251 cultures were fixed in a solution of 1:1 acetone-methanol for 15 min at − 20 °C. For primary glioblastoma cultures, cells were pre-incubated with primary rabbit anti-glial fibrillary acidic protein (GFAP) antibody (Dako, cat no. Z0334, RRID: AB_10013382) diluted 1:400 in Phosphate Buffered Saline (PBS; Thermo Fisher Scientific Australia, cat no. 14190144) overnight at 4 °C, followed by secondary goat anti-rabbit antibody conjugated to Texas Red-X (Thermo Fisher Scientific Australia, cat no. T-862, RRID: AB_2556781) diluted 1:200 in PBS for 2 h at room temperature. Cell nuclei were subsequently stained with 5 μM of DAPI (Thermo Fisher Scientific Australia, cat no. D1306) for 1 h at room temperature. Cells were washed in triplicate with PBS after each staining step and mounted on glass slides with Dako fluorescence mounting medium (Dako, cat no. S3023). For U251 cells, cell nuclei were stained with DAPI for 1 h at room temperature and subsequently mounted.

### Tumour cell quantification

Cells were imaged with a Nikon Ti-E inverted motorised fluorescence microscope with a sCMOS Andor Zyla camera at 20 × objective (Monash Micro Imaging – Alfred Research Alliance, Melbourne, Australia). For each sample/passage and treatment group, the total number of viable tumour cells – GFAP + /DAPI + and DAPI + cells for patient glioblastoma cultures and U251 cells, respectively – were quantified over 16 random fields. All images were analysed using the ImageJ image processing software (version 2.9.0/1.53t)^64^.

### Lactate dehydrogenase (LDH) cytotoxicity assay

Lactate dehydrogenase (LDH) levels in primary glioblastoma culture supernatants were quantified using the Roche colorimetric LDH Cytotoxicity Detection Kit (Roche, cat no. 11644793001), as per manufacturer’s instructions. Briefly, cell-free supernatants were incubated with LDH reaction mixture at a 1:1 ratio for 25 min at room temperature. Absorbance was measured at 492 nm using the FLUOstar® Omega Plate Reader (BMG LABTECH). Absorbance was directly proportional to supernatant LDH levels.

### Statistical analysis

All statistical analyses were conducted using the Graphpad Prism software (version 9). Normality of datasets was assessed via Q–Q plots. A one-way repeated measures ANOVA or one-way ANOVA with Brown-Forsythe and Welch corrections was utilised for experiments on patient samples and U251 cells, respectively. Post-hoc analyses included the Tukey’s HSD and Dunnett’s multiple comparisons tests. Data were expressed as mean ± SEM. An alpha level of 0.05 was set as the significance level. The mean difference and corresponding 95% confidence interval (CI) were reported where necessary.

## Supplementary Information

Supplementary Figure.

## Data Availability

The data supporting the findings of this study are available from the corresponding author, Dr Mastura Monif, upon request.
