# Supplementary material for: P2X7 receptor antagonism by AZ10606120 significantly reduced in vitro tumour growth in human glioblastoma
Source: Sci Rep. 2023 May 24;13:8435. doi: 10.1038/s41598-023-35712-5 (PMC10209214; doi:10.1038/s41598-023-35712-5)
Supplement: Supplementary file 1 — Supplementary Figure. [file 41598_2023_35712_MOESM1_ESM.pdf]

Supplementary Figure for the manuscript titled '***P2X7 receptor antagonism by AZ10606120 significantly reduced in vitro tumour growth in human glioblastoma***' for submission to *Scientific Reports*

Authors: Liyen K. Kan, Matthew Drill, Padmakrishnan C. Jayakrishnan, Richard P. Sequeira, Emily Galea, Marian Todaro, Paul G. Sanfilippo, Martin Hunn, David A. Williams, Terence J. O'Brien, Katharine J. Drummond and Mastura Monif

Supplementary Figure 1

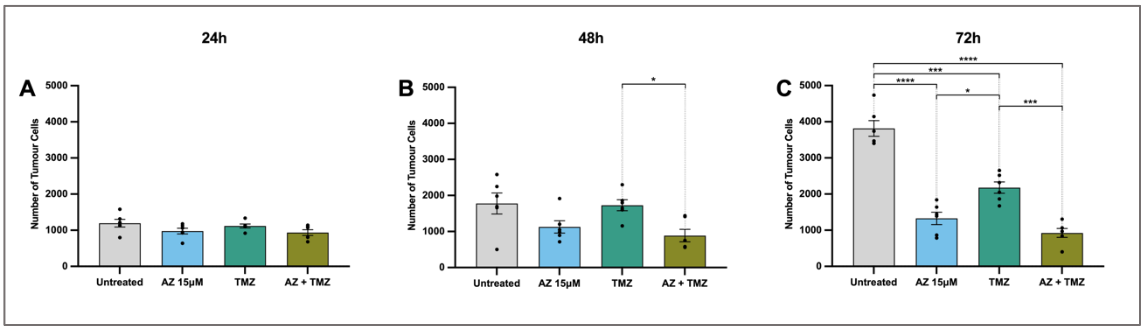

Supplementary Figure 1. The effects of P2X7 receptor (P2X7R) antagonism by AZ10606120 (AZ) compared to conventional temozolomide (TMZ) chemotherapy on the U251 human glioblastoma cell line at 24, 48 and 72 hours. U251 human glioblastoma cells were cultured and treated at 80% confluency with 15µM AZ, 50µM TMZ and a combination of AZ+TMZ for 24, 48 or 72 hours. The control groups were untreated cells. U251 cultures were counterstained with DAPI for 1 hour at room temperature. Images were acquired with a Nikon Ti-E inverted fluorescence motorised microscope equipped with a sCMOS Andor Zyla camera at 20× objective. The total number of DAPI+ cells were quantified across 16 random fields for each sample and treatment group. **A)** 24 hours post-treatment. Results were yielded from a one-way ANOVA. N=6 replicates, F(3, 16.82)=2.21, p=0.12. Untreated versus AZ 15µM: mean difference=217, 95% CI=-211.9 to 645.9; Untreated versus TMZ: mean difference=76.33, 95% CI=-335.1 to 487.8; Untreated versus AZ+TMZ: mean difference=258.8, 95% CI=-170.2 to 687.9; AZ 15µM versus TMZ: mean difference=-140.7, 95%CI=--453.6 to 172.3. AZ 15µM versus AZ+TMZ: mean difference=41.83, 95% CI=-315.1 to 398.7; TMZ versus AZ+TMZ: mean difference=182.5, 95% CI=-130.6 to 495.6. **B)** 48 hours post-treatment. Results were yielded from a one-way ANOVA with Dunnett's post-hoc. N=6 replicates, F(3, 14.72)=4.67, p=0.02. Untreated versus AZ 15µM: mean difference=651.3, 95% CI=-486.8 to 1789, p=0.37; Untreated versus TMZ: mean difference=50.17, 95% CI=-1057 to 1157, p>0.99; Untreated versus AZ+TMZ: mean difference=891.8, 95% CI=-250.6 to 2034, p=0.14; AZ 15µM versus TMZ: mean difference=-601.2, 95%CI=-1333 to 131, p=0.13. AZ 15µM versus AZ+TMZ: mean difference=240.5, 95% CI=-539.2 to 1020, p=0.89; TMZ versus AZ+TMZ: mean difference=841.7, 95% CI=103.5 to 1580, *p=0.02. **C)** 72 hours post-treatment. Results were yielded from a one-way ANOVA with Dunnett's post-hoc. N=6 replicates, F(3, 17.2)=56.55, p<0.0001. Untreated versus AZ 15µM: mean difference=2490, 95% CI=1583 to 3396, ****p<0.0001; Untreated versus TMZ: mean difference=1637, 95% CI=762.1 to 2512, ***p=0.001; Untreated versus AZ+TMZ: mean difference=2891, 95% CI=2052 to 3730, ****p<0.0001; AZ 15µM versus TMZ: mean difference=-852.3, 95%CI=-1595 to -109.4, *p=0.02. AZ 15µM versus AZ+TMZ: mean difference=401.7, 95% CI=-288.9 to 1092, p=0.38; TMZ versus AZ+TMZ: mean difference=1254, 95% CI=604.9 to 1903, ***p=0.0008. Error bars represent SEM.
